# Supplementary material for: Modulation of metabolic functions through Cas13d-mediated gene knockdown in liver
Source: Protein Cell. 2020 Mar 18;11(7):518–24. doi: 10.1007/s13238-020-00700-2 (PMC7095259; doi:10.1007/s13238-020-00700-2)
Supplement: Supplementary file 1 — Electronic supplementary material 1 (PDF 14505 kb) [file 13238_2020_700_MOESM1_ESM.pdf]

Materials and methods

Mice

8 weeks old male C57BL/6 (SLAC laboratory) mice and male heterozygous Ai9 (Rosa-CAG-LSL-tdTomato-WPRE, Jackson Laboratory) mice were used for Hydrodynamic tail vein injection. The use and care of animals complied with the guideline of the Biomedical Research Ethics Committee of Shanghai Institutes for Biological Science, Chinese Academy of Sciences.

Generation of plasmids

The CasRx and sgRNA backbone sequences were synthesized by the HuaGene Company. Then we generated vectors: *CAG-CasRx-p2A-GFP*, *U6-BbsI-DRs-EF1α-mCherry* and *U6-BbsI-CMV-GFP.* The sgRNA sequences were inserted by BbsI restriction enzyme. To insert two sgRNA sequences, overlapped PCR were performed using primers in the table S2. Vectors *U6-BbsI-DRs-EF1α-mCherry-2A-Pcsk9* and *U6-BbsI-DRs-EF1α-mCherry-2A-lncLstr* were constructed for screen of sgPcsk9 and sglncLstr. Vector *PBL-U6-DR-sgPten*s*-DR-CAG-CasRx-p2A-GFP-PBR and CMV-PBase* were constructed for piggyBac-mediated transfer of CasRx and sgPtens to hepatocytes. Vector *ITR-EFS-CasRx-Flag-polyA-U6-DR-sgRNA*s*-DR-ITR* was constructed to generate AAV8. All of the primers were listed in the table S2.

Cell culture and transfection

N2a (Neuro-2a) and 293T cell lines were purchased from Stem Cell Bank, Chinese Academy of Sciences. N2a and 293T cell lines were cultured with DMEM (Gibco) supplemented with 10% fetal bovine serum (Gibco), 1% penicillin/streptomycin (Thermo Fisher Scientific) and 0.1 mM non-essential

amino acids (Gibco) in an incubator at 37 °C with 5% CO_2. When cells reached 90% confluence, 293T and N2a cells were passaged at a ratio of 1:6 to 24-well plates. After 12 hours, 1 μg/well plasmids were introduced into cells with Lipofectamine 3000 (Thermo Fisher Scientific) using the standard protocol. 48 hours after transfection, GFP and mCherry positive cells were sorted by BD FACS Aria II for RNA extraction. For transcriptome sequencing, 30ug plasmids expressing shRNA or CasRx with sgRNAs were transfected into 10cm dishes. Then 500k positive cells were sorted out to make a pool for sequencing.

RNA extraction and qPCR

The total RNA was extracted by adding 500 μL Trizol (Invitrogen), 200 μL chloroform to the cells. After centrifuge at 12000 rpm for 15 min at 4 ℃， the supernatant was transferred to a 1.5 mL RNase-free tube. Then isopropanol and 75% alcohol were used to precipitate and purify the RNA. The cDNA was prepared using HiScript Q RT SuperMix for qPCR (Vazyme, Biotech) according to manufacturer's instructions. qPCR reactions were performed with AceQ qPCR SYBR Green Master Mix (Vazyme, Biotech). All of the reagents were precooled in advance. All of the primers used for qPCR reactions were listed in the table S3.

Preparation of AAV8

AAV plasmids along with adenoviral helper and AAV rep gene, AAV8 cap gene were introduced to HEK293FT cells by Polythylenimine Max (PEI, Polysciences) in DMEM+10% FBS media. Viral supernatant was harvested 7 days later, purified by iodixanol density gradient purification.

Hydrodynamic tail vein injection and hepatocyte isolation

Male C57BL/6 (SLAC laboratory) mice or heterozygous Ai9 (Rosa-CAG-LSL-

tdTomato-WPRE, Jackson Laboratory) mice at the age of 8 weeks were used for hydrodynamic tail vein injection. Mice were infected with 1×10^{11} transducing units (TU) of AAV in 100 μL PBS by intravenous injection. Vectors for hydrodynamic tail-vein injection were prepared using the Plasmid DNA purification Midi Kit (MACHEREY-NAGEL). For hydrodynamic liver injection, plasmid DNA suspended in 2 ml saline was injected via the tail vein in 5–7 s into 8-week-old male mice. Mice were sacrificed for analysis at 1, 4, 8, 15 or 16 days post-injection of plasmids or 3 weeks post-injection of AAV. Mouse primary hepatocytes were isolated by standard two-step collagenase perfusion and purified by 40% Percoll (Sigma) at low-speed centrifugation (1000 rpm, 10 min). Hepatocytes were resuspended in DMEM plus 10% fetal bovine serum (FBS) for FACS, and specific cell populations were used for RNA extraction or western blot analysis.

Immunofluorescence staining and western blot

Mice were sacrificed at 4 days post-injection of PBase plasmids and CasRx/sgPten plasmids. Separated liver lobes were harvested and fixed in 4% of paraformaldehyde overnight, and stored in 30% sucrose for 12h. After embedding in OCT compound (Sakura Finetek), 10μm liver sections were used for immunofluorescence staining. The following antibodies were used: anti-Pten (Cell Signaling, 9559, 1:100), anti-pAkt S473 (Cell Signaling, 4060, 1:100). For immunoblotting, proteins were separated by SDS-PAGE, then transferred onto a PVDF membrane and identified by immunoblot analysis with appropriate primary antibodies, including GAPDH antibody (Proteintech, 10494-1-AP, 1:5000), PTEN antibody (1:2000), p-AKT S473 antibody (1:2000), AKT antibody (1:2000), PCSK9 (10007185, 1:2000). HRP-labeled Goat Anti-Rabbit IgG (H+L) (1:1000) were from Beyotime Institute of Biotechnology. The protein bands were visualized with a BeyoECL Star Kit according to the manufacturer's instructions (Beyotime Institute of Biotechnology, P0018A).

Serum cholesterol and PCSK9 protein mensuration, serum biochemistry

Before whole blood collection, mice were fasted for 4 hours. Then whole blood was collected at the time of euthanasia. Whole blood were stood at room temperature for 1 hour and centrifuged at 2000 g for 20 min. Then transfer the serum into new tubes for further analysis. Serum PCSK9 protein and cholesterol were measured with Mouse Proprotein Convertase9/PCSK9 Quantikine ELISA Kit (R&D Systems) and the Infinity Cholesterol Reagent (Thermo Fisher Scientific), respectively, according to the manufacturer's instructions. In the CCl_4 group, WT mice were intraperitoneally injected with CCl_4 (1 mL/kg body weight; Shanghai Hushi, Shanghai, China) and sacrificed for analysis at 24 hours after injection. Mice intraperitoneally injected with the same volume of saline were sacrificed for analysis at 24 hours after saline injection. Serum parameters of liver functions, including alanine aminotransferase (ALT), aspartate transaminase (AST), were measured by automatic biochemical analyser at the Adicon Clinical Laboratories.inc (Shanghai, China).

RNA-seq analysis

The transcriptome libraries were sequenced using 150 bp paired-end Illumina Xten platform. After filtering the low-quality reads with SolexaQA (V3.1.7.1) , RNA-seq reads were aligned the reads to mm10 reference genome with Hisat2 (V2.0.4) . All uniquely mapped reads were used to calculate the read counts with htseq-count (v0.11.2). DEseq2 (1.24.0) were used to calculate differentially expressed genes. Genes with fold-change>2 and FDR<0.05 were treated as differentially expressed genes.

Statistical analysis

All values are reported as mean ± SD. A two-tailed unpaired Student's t-test

was used and statistical significance was determined as $*p < 0.05$, $**p < 0.01$, $***p < 0.001$. Graphpad Prism 8 was used for all statistical analysis.

Data availability

RNA-seq data are available with the SRA accession number SRP218720.

Supplementary figure legends

Fig. S1: Design of sgRNAs.

A. 10 sgRNAs designed in coding sequence of Pten. sgPten-5 (g5, red) and sgPten-6 (g6, red) were selected for in vivo targeted knockdown of Pten.

B. 11 sgRNAs designed in coding sequence of Pcsk9. sgPcsk9-5 (g5, red) and sgPcsk9-6 (g6, red) were selected for in vivo targeted knockdown of Pcsk9.

C. 11 sgRNAs designed in lncLSTR locus for screening. sglncLstr-5 (g5, red) and sglncLstr-6 (g6, red) were selected for in vivo targeted knockdown of lncLSTR.

Fig. S2: CasRx-mediated knock-down of metabolism regulator genes *in vitro*

A. N2a cells were transfected with plasmids expressing CasRx and sgRNAs. Left, design of plasmids used for *Pten* sgRNA screen. Right, mRNA levels of *Pten* were quantified in N2a cells receiving CasRx and indicated sgRNA (n=4).

B. Schematic of plasmids used for CasRx-mediated *Pten* knockdown in N2a cells with the combination of different sgRNAs.

C, D. Knockdown of *Pten* by combination of different sgRNAs in N2a cells (n=3).

E, F. 293T cells were transfected with plasmids expressing CasRx, sgRNA and *Pcsk9* (**E**) or *lncLstr* (**F**). Knockdown efficiency of *Pcsk9* (**E**) or *lncLstr* (**F**) was quantified in 293T cells receiving indicated sgRNA (n=3, except for sglncLstr-9, n=2). Data are represented as mean with SD. **p<0.01.

Figure S3: CasRx-mediated knockdown of metabolic genes in hepatocytes *in vivo*

A. Plasmids expressing CasRx and sgRNAs were delivered to wild-type mouse livers by hydrodynamic tail-vein injection.

B. Hepatocytes receiving the plasmids encoding CasRx, sgPten-5 and sgPten-6 (GFP+/mCherry+) were purified to quantify the expression of *Pten* by qPCR (n=4). The expressions of *Pten* were not significantly changed in hepatocytes receiving CasRx and sgLacZ (n=3).

C, D. CasRx-mediated knockdown of *Pcsk9* in hepatocytes. *Pcsk9* mRNA levels and protein levels were quantified by qPCR (n=4) (**C**) and western blot (**D**). The expressions of *Pcsk9* were not significantly changed in hepatocytes receiving CasRx and sgLacZ (n=3) (**C**).

E, F. CasRx-mediated knockdown of *lncLstr* in hepatocytes. *lncLstr* (**E**) and its downstream gene *Cyp8b1* (**F**) were quantified by qPCR (n=4). The expressions of *lncLstr* were not significantly changed in hepatocytes receiving

CasRx and sgLacZ (n=3) (**E**).

G. CasRx with sgRNA arrays simultaneously knocked down *Pten*, *Pcsk9* and *lncLstr* in hepatocytes (n=5). Data are represented as mean with SD. *p<0.05, **p<0.01, ***p<0.001.

Fig. S4: Gradually loss of CasRx plasmids in hepatocytes.

A. The proportions of hepatocytes expressing indicated fluorescence proteins were quantified by flow cytometry at day 1, 4, 8 and 15 after hydrodynamic tail-vein injection of CasRx plasmids into ROSA-CAG-lsl-tdTomato mice (D1, n=4; D4, D8, D15, n=3). Data are represented as mean with SD.

Table S1.sgRNA sequences

pten sg1	ggatcagagtcagtggtgtcagaatatcta
pten sg2	ggttcattgtcactaacatctggagtcaca
pten sg3	gaagttgaactgctagcctctggatttgat
pten sg4	ggctcctctactgtttttgtaaagtatagt
pten sg5	ggagagaagtatcggttggccttgtctttg
pten sg6	ggtgagtacaagatactccttgtcattatc
pten sg7	gcacgctctatactgcaaatgctatcgatt
pten sg8	gagcatcttgttctgtttgtggaagaactc
pten sg9	gatatcaccacacacaggcaatggctgagg
pten sg10	gaattggaggaatatatcttcacctttagc
pcsk9 sg1	gcaccctggatgctggtatctaagagatac
pcsk9 sg2	gtgatggtgaccctgccctcaatctcccga
pcsk9 sg3	gtcccatcctcctccggcacgctgttgaag
pcsk9 sg4	gtctgtggaagcgtgtcccatcctcctccg
pcsk9 sg5	gtgggtgccgtggctgtcacacttgctcgc
pcsk9 sg6	cagcatcccggccgctgaccacacctgcca
pcsk9 sg7	ggctgtgcaggctggtgcccttggccacac
pcsk9 sg8	gtgcccttcccttgacagttgagcacacgc
pcsk9 sg9	ggagtagaggcaggcgtcgtcccggaagtt

pcsk9 sg10	gattagctgactcttccgaataaactcca
pcsk9 sg11	gccggcaggcagcgttgaggatgcggctat
lstr sg1	gtttcaggttccagcgacttccattgcaaa
lstr sg2	gatcagatagaatgttcctcaaggtattgg
lstr sg3	cagcttgctttacccaagcacctgtctcaa
lstr sg4	gttgtactgtcttctctgtgggcagggtat
lstr sg5	gtatacagaactagagcaaggtcacctagcc
lstr sg6	gagtcactgcttcagcctttactaacttca
lstr sg7	gtctaaccccagtgctagaactggattctg
lstr sg8	gtctctttggcctatggctctctgataagg
lstr sg9	gtacttgtgcacacacaggtgggcatatgc
lstr sg10	ggcaagagccacacatttagcttcattaac
lstr sg11	ggctttatacatgtagcaatcgtcttgtct
lacz sg	cgtctggccttcctgtagccagctttcatc
shPTEN-5	acaaggccaaccgatacttctCTCGAGagaagtatcggttggccttgtTTTTTG
shPTEN-6	atgacaaggagtatcttgtacCTCGAGgtacaagatactccttgtcatTTTTTG

Table S2. **Primers used for plasmids construction**

Pten sg5+6-R1,R2	gaagacccccttggataatgacaaggagtatcttgtactcaccgtttcaaacccc
LSRT sg5+6 F1	cacctagcccaagtaaacccctaccaactggtcggggtttgaaacgagtcact
LSRT sg5+6 R1,R2	gaagacccccttgtgaagttagtaaaggctgaagcagtgactcgtttcaaacccc
LSRT sg5+6 F2	gaagacctaaacgtatacagaactagagcaaggtcacctagcccaagtaaaccc
pcsk9 sg5+6 F1	acttgctcgccaagtaaacccctaccaactggtcggggtttgaaaccagcatc
pcsk9 sg5+6 R1,R2	gaagacccccttgtggcaggtgtggtcagcggccgggatgctggtttcaaaccccg
pcsk9 sg5+6 F2	gaagacctaaacgtgggtgccgtggctgtcacacttgctcgccaagtaaacc
Pten sg5+6 pcr F1	caggtctcactagtgagggcctatttcccatgat
Pten sg5+6 pcr R1	caggtctcatcagaaataccgcatcagaattcaaa
Lstr sg5+6 pcr F	caggtctcactgagagggcctatttcccatgatt
Lstr sg5+6 pcr R	caggtctcacttcaaataccgcatcagaattcaaa
2A-F	gcggcatggacgagctgtacaagggcagtgga
2A-R	cgacgtcaccgcatgttagcagacttcctctgccctctccactgcccttgtacagc

Lstr-F	ctaacatgcggtgacgtcgaggagaatcctggcccaagtctgtcaaactgaatggg
Lstr-R	taaacaagttttacttgtagcttatcaatccaatgcaccattttactccaatatggt
Pcsk9-F	ctaacatgcggtgacgtcgaggagaatcctggcccagatggaagcagccaggtg
Pcsk9-R	taaacaagttttacttgtatcactctggagcagaagctgggg
pten-sg5+6 pcr F2	gattcgacattgattattgagatgggctggccttttgctcag
pten-sg5+6 pcr R2	aattgattactattaataactagtcaataatcaatgtccgtaaggagaaaataccgcat

Table S3. qPCR primers

mouse Pten qPCR F	tggattcgacttagacttgacct
mouse Pten qPCR R	gcggtgtcataatgtctctcag
mouse Pcsk9 qPCR F	gcccatcgggagattgagg
mouse Pcsk9 qPCR R	ttcccttgacagttgagcaca
mouse lncLSTR qPCR F	caggtgcttgggtaaagcaa
mouse lncLSTR qPCR R	taggaggcagagcttgcttg
mouse Gapdh F	ccgtagacaaaatggtgaaggt
mouse Gapdh R	cgtgagtggagtcatactggaa
human Gapdh F	gtggacctgacctgccgtct
human Gapdh R	ggaggagtgggtgtcgctgt
mouse Actb qpcr F	gtgacgttgacatccgtaaaga
mouse Actb qpcr R	gccggactcatcgtactcc
mouse Fasn qpcr F	ggaggtggtgatagccggtat
mouse Fasn qpcr R	tgggtaatccatagagcccag
mouse Srebf1 qpcr F	gcagccaccatctagcctg
mouse Srebf1 qpcr R	cagcagtgagtctgccttgat
mouse G6pc qpcr F	cgactcgctatctccaagtga
mouse G6pc qpcr R	gggcgttgtccaaacagaat
mouse PEPCK qpcr F	ctgcataacggtctggacttc
mouse PEPCK qpcr R	gccttccacgaacttcctcac
mouse Cyp8b1 qpcr F	cacggggatgtcttcacgg
mouse Cyp8b1 qpcr R	tgagcaccagttcttttgcatag

Table S4. Antibodies

anti-PTEN	Cell Signaling	Cat# 9559
anti-pAKT S473	Cell Signaling	Cat# 4060
anti-AKT	Cell Signaling	Cat# 9272
anti-GAPDH	Proteintech	Cat# 10494-1-AP
PCSK9 (human) Polyclonal Antibody	Cayman	Cat# 10007185

HRP-labeled Goat Anti-Rabbit IgG (H+L)	Beyotime	Cat# A0208
Cy3-conjugated AffiniPure Donkey Anti-Rabbit IgG (H+L)	Jackson	Cat# 711-165-152

Table S5. Software and Algorithms

SolexaQA v3.1.7.1	Murray P Cox et al.,2010	https://sourceforge.net/projects/solexaqa/
hisat2 v2.0.4	Daehwan Kim et al.,2015	https://ccb.jhu.edu/software/hisat2/index.shtml
Deseq2 1.24.0	Michael I Love et al.,2014	https://bioconductor.org/packages/release/bioc/html/DESeq2.html
Htseq v0.11.2	Simon Anders et al.,2015	https://htseq.readthedocs.io/en/release_0.11.1/count.html

Figure S1

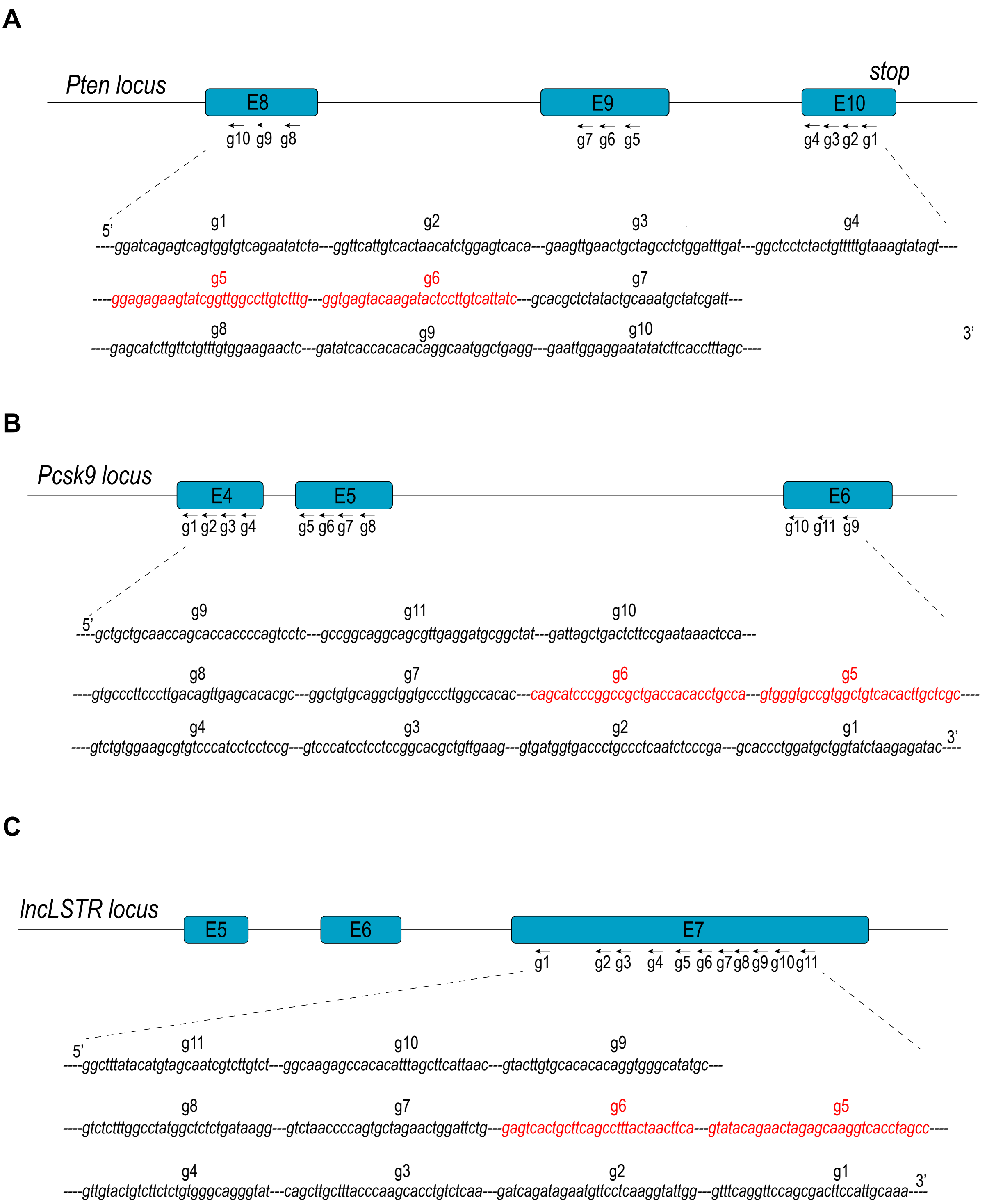
A
Pten locus
stop
E8
E9
E10
g10 g9 g8
g7 g6 g5
g4 g3 g2 g1
5'
g1
g2
g3
g4
----ggatcagagtcagtggtgtcagaatatcta---ggttcattgtcactaacatctggagtcaca---gaagttgaactgctagcctctggatttgat---ggctcctctactgtttttgtaaagtatagt----
g5
g6
g7
----ggagagaagtatcggttggccttgtctttg---ggtgagtacaagatactccttgtcattatc---gcacgctctatactgcaaatgctatcgatt---
g8
g9
g10
3'
----gagcatcttgttctgtttgtggaagaactc---gatatcaccacacacaggcaatggctgagg---gaattggaggaatatatcttcacctttagc----
B
Pcsk9 locus
E4
E5
E6
g1 g2 g3 g4
g5 g6 g7 g8
g10 g11 g9
5'
g9
g11
g10
----gctgctgcaaccagcaccaccccagtcctc---gccggcaggcagcgttgaggatgcggctat---gattagctgactcttccgaataaactcca---
g8
g7
g6
g5
----gtgcccttcccttgacagttgagcacacgc---ggctgtgcaggctggtgcccttggccacac---cagcatcccggccgctgaccacacctgcca---gtgggtgccgtggctgtcacacttgctcgc----
g4
g3
g2
g1
3'
----gtctgtggaagcgtgtcccatcctcctccg---gtcccatcctcctccggcacgctgttgaag---gtgatggtgaccctgccctcaatctcccga---gcaccctggatgctggtatctaagagatac----
C
lncLSTR locus
E5
E6
E7
g1
g2 g3
g4
g5 g6 g7 g8 g9 g10 g11
5'
g11
g10
g9
----ggctttatacatgtagcaatcgtcttgtct---ggcaagagccacacatttagcttcattaac---gtacttgtgcacacacaggtgggcatatgc---
g8
g7
g6
g5
----gtctctttggcctatggctctctgataagg---gtctaaccccagtgctagaactggattctg---gagtcactgcttcagcctttactaacttca---gtatacagaactagagcaaggtcacctagcc----
g4
g3
g2
g1
3'
----gttgtactgtcttctctgtgggcagggtat---cagcttgctttacccaagcacctgtctcaa---gatcagatagaatgttcctcaaggtattgg---gtttcaggttccagcgacttccattgcaaa----

Figure S2

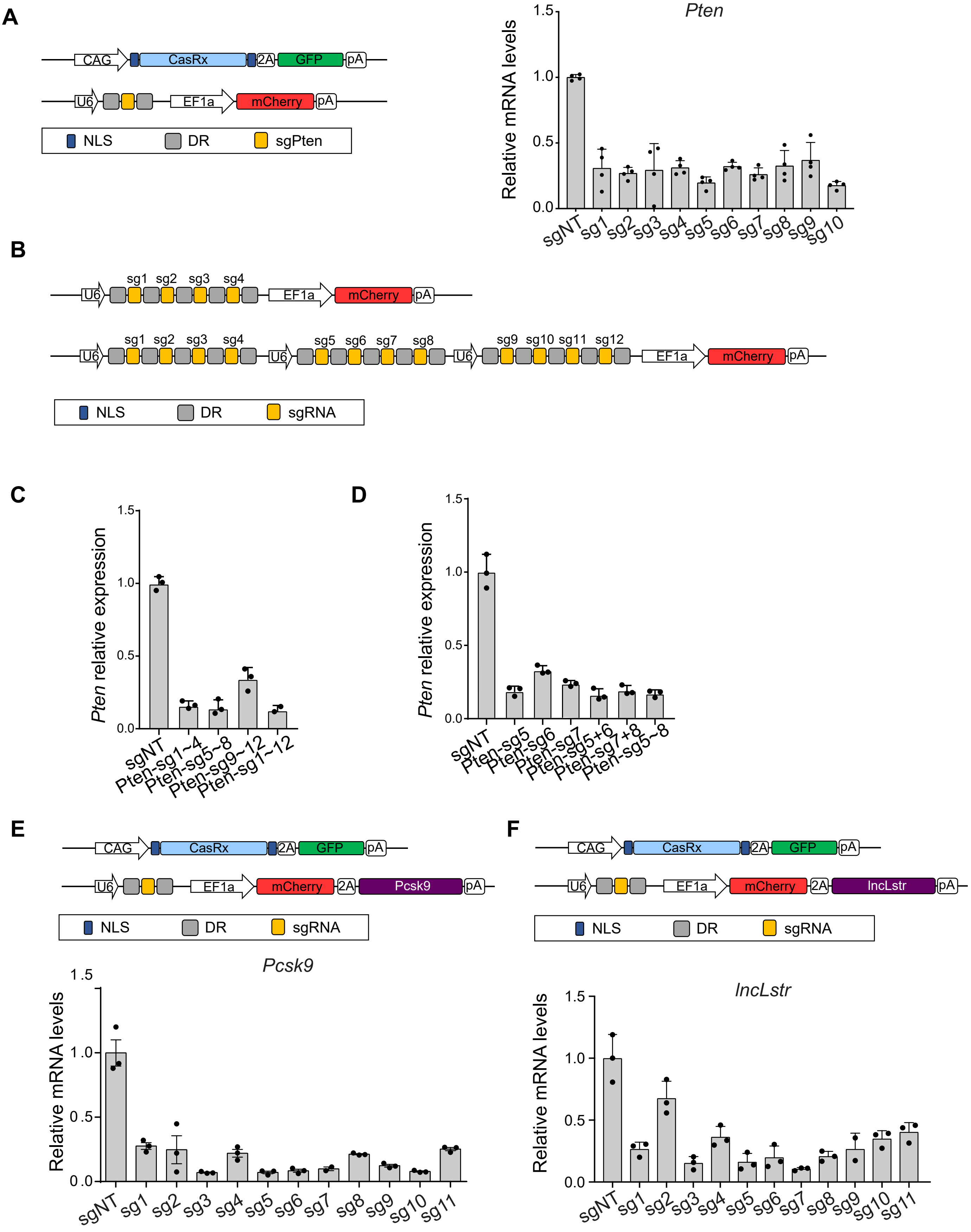
A
CAG
CasRx
2A
GFP
pA
U6
EF1a
mCherry
NLS
DR
sgPten
Pten
Relative mRNA levels
1.5
1.0
0.5
0.0
sgNT
sg1
sg2
sg3
sg4
sg5
sg6
sg7
sg8
sg9
sg10
B
sg1 sg2 sg3 sg4
sg5 sg6 sg7 sg8
sg9 sg10 sg11 sg12
U6
EF1a
mCherry
pA
NLS
DR
sgRNA
C
Pten relative expression
sgNT
Pten-sg1~4
Pten-sg5~8
Pten-sg9~12
Pten-sg1~12
D
Pten relative expression
sgNT
Pten-sg5
Pten-sg6
Pten-sg7
Pten-sg5+6
Pten-sg7+8
Pten-sg5~8
E
CAG
CasRx
2A
GFP
pA
U6
EF1a
mCherry
2A
Pcsk9
pA
NLS
DR
sgRNA
Pcsk9
Relative mRNA levels
sgNT
sg1
sg2
sg3
sg4
sg5
sg6
sg7
sg8
sg9
sg10
sg11
F
CAG
CasRx
2A
GFP
pA
U6
EF1a
mCherry
2A
lncLstr
pA
NLS
DR
sgRNA
lncLstr
Relative mRNA levels
sgNT
sg1
sg2
sg3
sg4
sg5
sg6
sg7
sg8
sg9
sg10
sg11

Figure S3

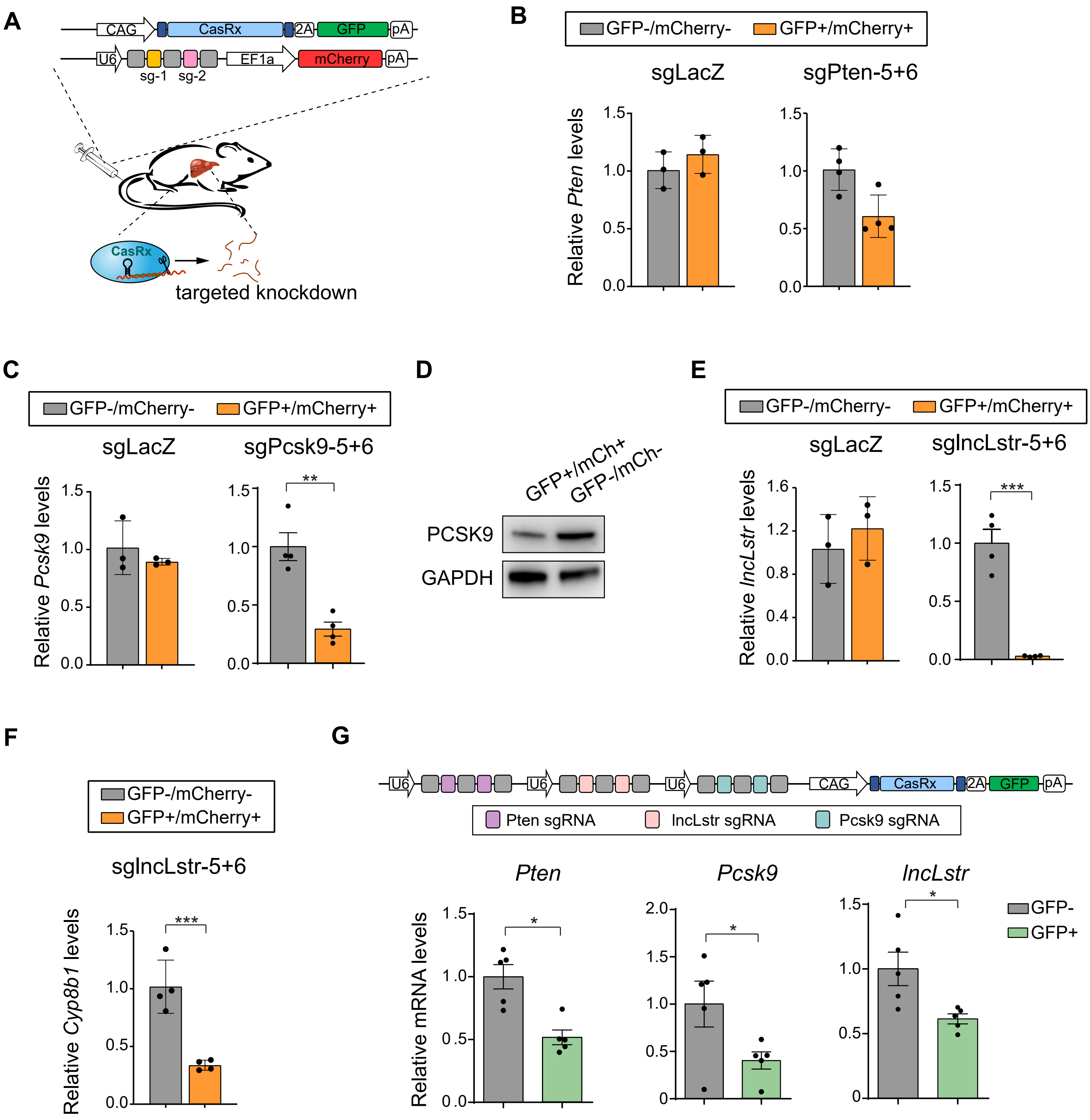

A
CAG
CasRx
2A
GFP
pA
U6
sg-1
sg-2
EF1a
mCherry
pA
CasRx
targeted knockdown
B
GFP-/mCherry-
GFP+/mCherry+
sgLacZ
sgPten-5+6
Relative Pten levels
C
GFP-/mCherry-
GFP+/mCherry+
sgLacZ
sgPcsk9-5+6
Relative Pcsk9 levels
**
D
GFP+/mCh+
GFP-/mCh-
PCSK9
GAPDH
E
GFP-/mCherry-
GFP+/mCherry+
sgLacZ
sglncLstr-5+6
Relative lncLstr levels
***
F
GFP-/mCherry-
GFP+/mCherry+
sglncLstr-5+6
Relative Cyp8b1 levels
***
G
U6
U6
U6
CAG
CasRx
2A
GFP
pA
Pten sgRNA
lncLstr sgRNA
Pcsk9 sgRNA
Pten
Pcsk9
lncLstr
Relative mRNA levels
GFP-
GFP+

Figure S4

A

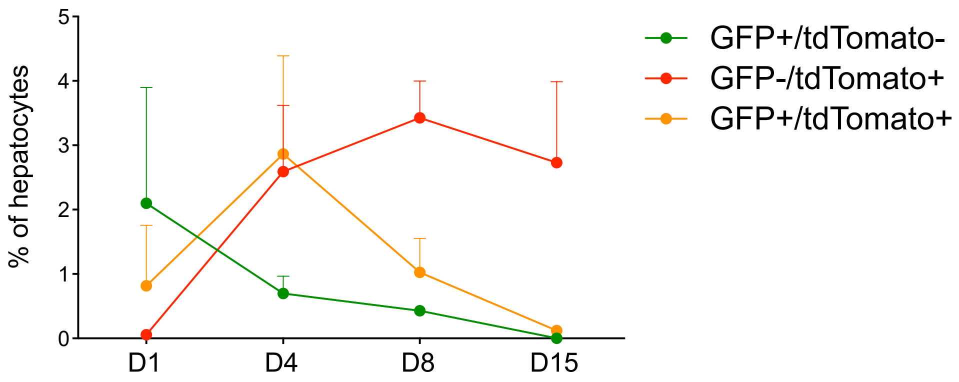

% of hepatocytes
5
4
3
2
1
0
D1
D4
D8
D15
GFP+/tdTomato-
GFP-/tdTomato+
GFP+/tdTomato+
